# Genetic and environmental links between traits of cocoa beans and pods clarify the phenotyping processes to be implemented

**DOI:** 10.1038/s41598-020-66969-9

**Published:** 2020-06-18

**Authors:** Fabien Doaré, Fabienne Ribeyre, Christian Cilas

**Affiliations:** 1 CIRAD, UPR Bioagresseurs, F- 97387 Kourou, Guyane, France. Bioagresseurs, Univ Montpellier, CIRAD, Montpellier, Kourou, France; 2 CIRAD, UPR Bioagresseurs, F-34398 Montpellier, France. Bioagresseurs, Univ Montpellier, CIRAD, Montpellier, France

**Keywords:** Genetics, Plant sciences

## Abstract

The average weight of cocoa beans is not generally taken into account during breeding processes, although it is a trait of interest. Several studies indicate that the weight of the beans has a high heritability in *Theobroma cacao*. However, the values obtained from different countries for the same clone often vary. In this study, we analyzed the effect of different factors on the weight of the beans. Apart from the clone effect, three main factors had an impact: i) the number of beans per pod: a good filling of the pod with beans tended to limit the weight of the beans, ii) the position of the beans in the pod: beans in the apical part of the pod were significantly lighter than the others and iii) the longer the duration of the fructification cycle the heavier the beans were (positive genetics correlation). These results lead us to propose protocols aimed at normalizing the phenotypic values of the genetic material. To obtain a reliable estimate of the bean weight, the following is proposed: either to use beans obtained from manual pollination to saturate the pods with beans, or to systematically use the number beans in the pods as a covariable.

## Introduction

The cacao tree (*T. cacao*) is a small, evergreen tree native to the Amazonian forest^1^. This tree of the Malvaceae family (formerly Sterculiaceae) is grown for its beans contained in the fruits. The cacao tree’s growth is indeterminate and its fruit production can vary from 20 to over 100 years, with fruit growing on both trunk and branches. The fruits, known as cacao pods, arise from the pollination of flowers grouped in flower cushions^2^. The pods contain beans, which are extracted from the pod and fermented with surrounding mucilage, then dried to produce fermented dried cocoa, the raw material used to make chocolate. The production of commercial cocoa beans from one tree over a given period depends on the number of pods produced, the number of seeds per pod and the mean seed weight^3^.

The main goals of cocoa breeding are to increase the cocoa production per tree and to improve the cocoa quality. To increase the production per tree implies: i) improved resistance to the pest and diseases of cocoa, ii) increased pods produced per tree, iii) increased number of beans per pod and iv) increased average bean weight.

In fact, the production Yi of a tree i between date t and date t + s, (Yi [t, t + s]) can be written as:

$${\rm{Y}}{\rm{i}}=[{\rm{t}},{\rm{t}}+{\rm{s}}]={\rm{N}}{\rm{o}}{\rm{P}}{\rm{o}}{\rm{d}}{\rm{s}}{\rm{i}}[{\rm{t}},{\rm{t}}+{\rm{s}}]{\rm{x}}{\rm{N}}{\rm{o}}{\rm{S}}{\rm{P}}{\rm{i}}[{\rm{t}},{\rm{t}}+{\rm{s}}]{\rm{x}}{\rm{S}}{\rm{W}}{\rm{i}}[{\rm{t}},{\rm{t}}+{\rm{s}}]$$

where:

NoPodsi [t, t + s]: number of healthy pods produced by tree i between date t and date t + s.

NoSPi [t, t + s]: mean number of seeds per pod for tree i between date t and date t + s.

SWi [t, t + s]: mean seed weight for tree i between date t and date t + s.

The heritability of the number of healthy pods produced per tree is relatively weak (<0.4), which indicates that this trait is mostly dependent on environmental factors^3–5^. Actually, the trait is the result of several elementary traits or processes: flowering, pollination, fruit-setting, cherelle wilt, diseases and insect attacks^6–8^. In addition, these elementary traits, with generally low heritabilities, are instead dependent on environmental conditions, like soil and climate.

The number of seeds per pod and the mean seed weight are traits that should be also improved to increase production per tree. The number of seeds per pod depends on several factors, including the number of ovules per ovary, the fertility of the ovules, which varies according to the self-compatibility or self-incompatibility of the cacao genotype, and natural pollination conditions^9^. Mean seed weight is an important trait with high heritability^10^, a large bean size as well as uniformity of the bean size being the characteristics of interest for the chocolate industry. The average bean weight is therefore an essential trait to take into account in the genetic improvement of cocoa.

Several studies have revealed that the mean seed weight showed a normal distribution per clone and a high heritability (≥0.5)^10–12^, but the values obtained in different studies remain quite variable for the same clones. We wanted to understand how this trait varied according to several factors, including the filling of pods with beans, and the position of the beans in the pods. Observations on several clones in the CIRAD collection in French Guiana were conducted to answer these questions: i) what is the impact of filling the pods with beans on the weight of the beans? ii) is there a heterogeneity in the size of the beans in the pods (between the peduncular, medial and apical pod sectors)? what is the link with other factors like the duration of the fruiting cycle (from pollination to maturation)? This is the first time (as far as we are aware) that these criteria (number of beans per pod, position of the bean in the pod, duration of the fruiting cycle) have been taken into account to explain the bean weight, in addition to the genetic effect with the study of several clones.

## Material and methods

### Plant material

Seven clones situated in the Pointe-Combi experimental station of CIRAD in French Guiana were used for this study (ELP 35 A, GF 23, CCN 51, ICS 60, IMC 97, NA 79, PA 121).

ELP 35 A: clone collected in the area of Crique Euleupoussing in the French Guiana forest (Guiana group)

GF 23: clone collected in French Guiana coast (Amelonado group),

CCN 51: clone coming from a cross of the hybrid (IMC 67 x ICS 95) with an Ecuadorian cultivar locally known as “Canellos” (Hybrid),

ICS 60: clone coming from selections in Trinidadian farms, (Criollo group),

IMC 97: clone collected by Pound in Iquitos area (Peru), (Iquitos group),

NA 79: clone collected by Pound in Nanay area (Peru), (Nanay group),

PA 121: clone collected by Pound in Parinari area (Peru), (Marañon group).

These clones were chosen because of i) their good representativity of the genetic diversity of *T. cacao*^13^, ii) their availability of flowers and pods, and iii) their good physiological condition in the Pointe-Combi experimental station. Several trees per clone were available for the study, between 2 and 16 trees per clone, depending on the clone.

### Observations

**Figure 1:**
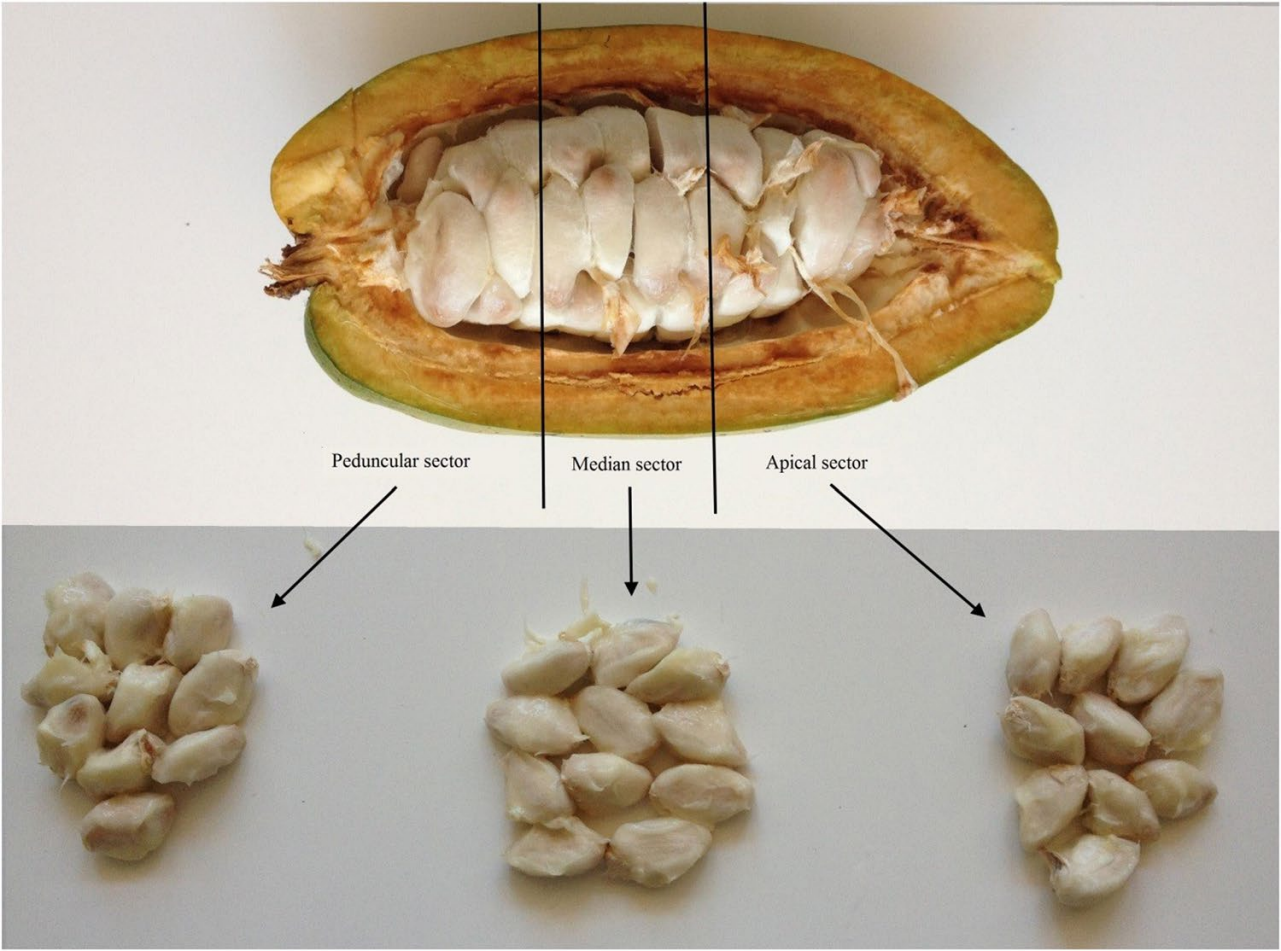
Study of the seeds according to the three sectors of the pod: Peduncular sector, Median sector, Apical sector.

The mature pods were harvested and taken to the CIRAD laboratory in Kourou (French Guiana) between February 2016 and March 2019. On the same day of harvesting, the following were measured: weight of the whole pod, weight of the wet beans and weight of the cortex. The beans of each pod were counted and separated into 3 sectors of equivalent size (Fig. 1): peduncular sector, median sector and apical sector. The beans from each sector were weighed and the average weight of a bean determined for each sector. In addition, fruiting cycle durations (FCD) – from pollination to pod maturation - were measured on a sample of manual pollination pods for each of the seven clones.

### Data analysis

For the number of beans per pod, the pod weights and the total weight of the beans, one way analyses of variance were performed with the factor “clone”. For pod weights and overall bean weight covariance analyses were also performed using the number of beans per pod as a covariate.

For the bean weight per sector, two way analyses of variance were performed (clone and sector) as well as covariance analyses using the number of beans per pod as covariate.

The broad sense heritability for these different traits was estimated, along with the associated confidence intervals. The confidence intervals were estimated by the Wald method^14^. Genetic and environmental correlations between the several pod and bean traits on one hand, and the duration of the fruiting cycle on the other hand were estimated using a multivariate general linear model (for the pods with the duration of the fruiting cycle data). Data analyses were performed with Proc GLM and Proc Mixed (SAS system)^15^.

## Results

### Comparison of clones and heritability values

The number of beans per pod had a moderate heritability (0.27). The other pods and beans traits were very different depending on the clones, with very strong heritabilities (Table 1), especially for the average weight of a bean (0.70).

**Table 1:** Comparison of clones for the number of beans per pod (*NBeans*) for the weight of a pod (*PodW*) in g, for the weight of the cortex (*CortexW*) in g, for the weight of beans per pod (*BeansW*) in g, for the average weight of a bean (*BeanWm*) in g, and multiple mean comparison test (Newman-Keuls 5%)*. For the last column, average weight of a bean adjusted to the covariate: number of beans per pod. Broad sense heritabilities (h²) of the traits with confidence interval at 5% (CI).

Clone	*NBeans*	*PodW*	*CortexW*	*BeansW*	*BeanWm*	*BeanWm Cov: NBeans*
IMC 97	44.9 a*	714.8 a	548.8 a	166.1 a	3.80 c	3.95 c
ICS 60	36.4 b	592.5 c	426.2 c	166.3 a	4.54 b	4.61 b
GF 23	34.0 b	312.2 d	238.0 d	74.2 b	2.24 e	2.27 e
NA 79	30.3 c	339.4 d	259.1 d	80.3 b	2.70 d	2.70 d
CCN 51	29.0 cd	659.9 b	495.7 b	164.2 a	5.64 a	5.63 a
ELP 35 A	27.2 d	351.1 d	283.1 d	68.0 b	2.51 d	2.47 d
PA 121	24.7 d	310.2 d	241.6 d	68.5 b	2.79 d	2.72 d
**h²	0.27	0.54	0.49	0.59	0.70	0.72
***CI	[0.04; 0.50]	[0.25; 0.82]	[0.21; 0.78]	[0.31; 0.86]	[0.47; 0.94]	[0.48; 0.95]

* means followed by the same letter are not significantly different at the 5% threshold by the Newman & Keuls test

**h²: broad sense heritability

*** CI: confidence intervals estimated by the Wald method

.

The number of beans per pod was significantly different among clones, from 24.7 beans per pod for PA 121 to 44.9 beans per pod for IMC 97 (Table 1). The clone IMC 97 had a significantly higher number of beans per pod than all the other clones tested. Three other homogeneous groups of clones were determined. ICS 60 and GF 23 had a higher number of beans than NA 79. This one had a higher number of beans than ELP 34 A and PA 121, with CCN 51 being between the two last groups. The differences among clones were significant for all the traits studied. IMC 97 also had higher values for total pod weight, cortex weight and total bean weight per pod (Table 1). On the other hand, CCN 51 clone had the highest average bean weight (Table 1). Indeed, for the average bean weight, mean values per clone ranged from 2.24 for GF 23 to 5.64 for CCN 51. Five groups were identified: CCN51 had the highest mean bean weight followed by ICS 60 that had a higher weight of a bean than IMC 97. GF23 had a significantly lower mean weight bean than all other clones.

**Figure 2:**
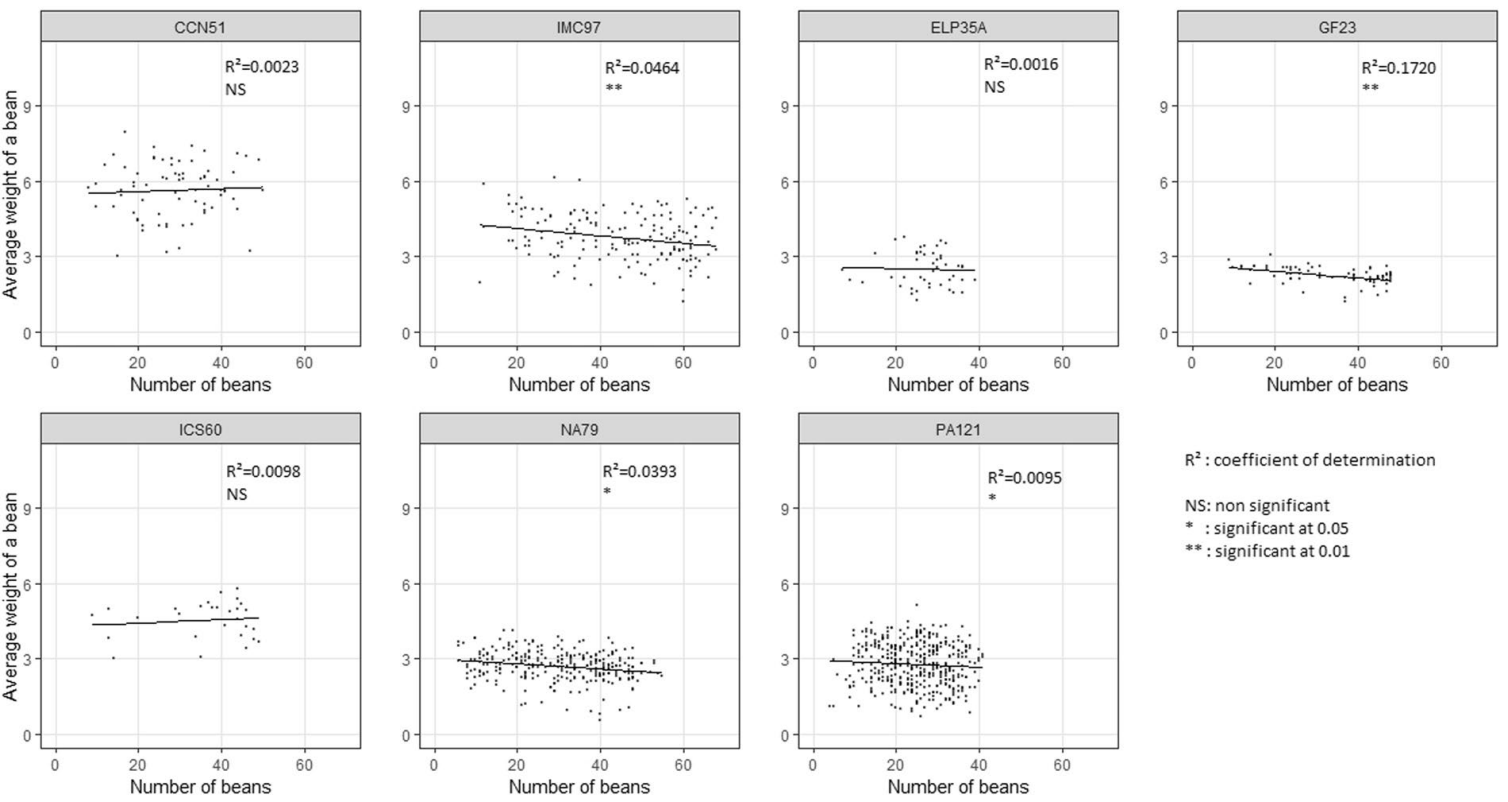
Regression between the average weight of a bean and the filling of the pods with beans for each of the 7 clones.

For the average bean weight, the covariance analysis indicated that there was an effect of the covariate: “number of beans per pod” (Table 2). Pods better filled with beans have lighter beans on average for most clones (Fig. 2). However, the means adjusted to this covariate were in the same order for the 7 clones considered (Table 1). To verify if the effect of the covariate was the same for each of the 7 clones, a regression of the average bean weight on the number of beans per pod was performed for each of the 7 clones (Fig. 2), and the regressions were significant for only 4 clones (IMC 97, GF 23, NA 79 and PA 121).

**Table 2:** General linear model for the mean weight of a bean according to clone and sector factors, and the covariate “number of beans per pod”.

Source	*DF	**Type III SS	Root mean square	*** F value	****Pr> F
Clone	6	2279.5	379.92	610.35	<0.0001
Sector	2	46.1	23.06	37.05	<0.0001
Clone*Sector	12	18.8	1.56	2.51	0.003
*NBeans*	1	31.9	31.91	51.27	<0.0001

* degrees of freedom.

** type III sums of squares (all effects are adjusted to all others).

*** value of the Fisher test

**** the significance value for the test.

### Sources of bean weight variability

The effect of the sectors (peduncle, median, apical) on the average bean weight was then taken into account. The clone and sector effects were very significant and the interaction between these two factors was also significant, but with a lesser effect (Table 2). The effect of the sector therefore existed whatever the clone.

Comparisons of clone and sector means were then made using this model (Table 3). The clone means were similar to those previously estimated (for instance, for CCN 51, the estimated bean weight was 5.64 g, 5.63 g taking into account the pod filling and 5.62 g taking into account also the pod sector), and therefore the clone ranking was not changed. For other clones, the differences between the several assessments were more important. There was a strong difference between the 3 sectors considered with a significantly lower bean weight mean in the apical pod region (reduction of 15% on average in comparison to the two other sectors), (Table 3).

**Table 3:** Comparison of clones means and sectors means, with cov: *NBeans*.

Clone	BeanWm	Sector	BeanWm
CCN 51	5.62 a*	Pedunc.	3.62 a
ICS 60	4.60 b	Median	3.56 a
IMC 97	3.88 c	Apic.	3.22 b
Pa 121	2.79 d		
Na 79	2.70 d		
ELP 35 A	2.48 e		
GF 23	2.25 f		

* means followed by the same letter are not significantly different at the 5% threshold by the Newman & Keuls test.

**Figure 3:**
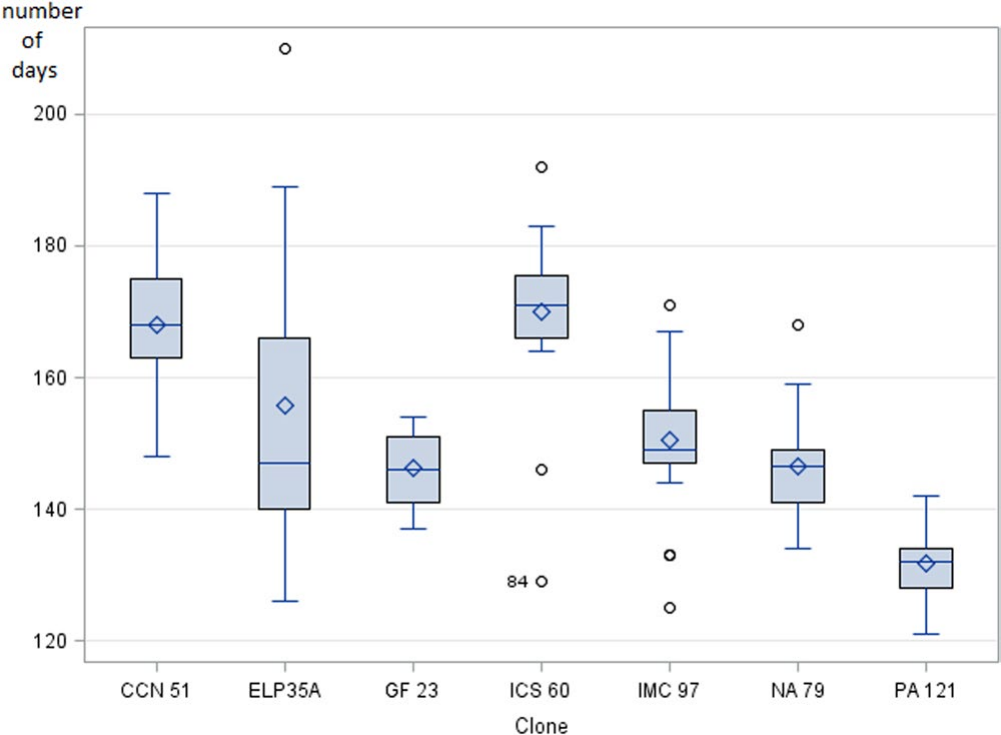
Box plot of fruiting cycle durations (FCD) of the seven clones (unit: number of days).

Fruit cycle durations (FCD) were also observed for each of the 7 clones studied and significant differences were detected, from 132 days for PA 121 to 170 days for ICS 60 (Fig. 3). An important variability was observed for the ELP 35 A clone; indeed, this clone did not show very clear signs of maturation with pods that remained partially green. FCD is genetically correlated to the pods and beans traits, particularly the beans weight (Table 4), with genetic correlations of 0.81 and 0.80 (with *BeansW* and *BeansWm* respectively). The longer the fruiting cycles of the clones, the heavier were the beans.

**Table 4:** Genetic correlations and residual correlations between the studied traits and fruit cycle duration (*FCD*).

*FCD*	*NBeans*	*PodW*	*CortexW*	*BeansW*	*BeanWm*
G correlation	0.46	0.69	0.63	0.81	0.80
R correlation	−0.05	0.01	0.03	−0.05	−0.04

## Discussion

There is a strong genetic effect on the weight of pods and beans, which confirms previous studies^3,10,16^. The effect of pod filling with beans on the average weight of a bean is highlighted; on average, for a given genotype, pods with more beans have slightly lighter beans. There would therefore probably be competition between beans within pods, i.e. beans may lack space for expansion when the pod contains many beans. However, this effect seems to be different depending on the genotypes; it is stronger for IMC 97 or GF 23 for instance (Fig. 2). These results lead us to propose protocols aimed at normalizing the phenotypic values of the genetic material. In order to obtain a reliable estimate of the bean weight, the following is proposed: i) either to use beans obtained from manual pollination to saturate the pods with beans, or ii) to systematically use the number beans in the pods as a covariable. By standardizing this measurement of bean weight, it will be easier to compare results from different laboratories.

The number of beans per pod depends on the number of ovules per ovary and the level of pollination of the flowers^6^. The number of ovules per ovary is a very heritable trait^3^; on the other hand, effective pollination depends on several factors: the self (in) compatibility of plant material^17^, the availability and intensity of pollinating insects traffic and therefore many environmental factors can have effects on the number of beans per pod^9,18^.

The average weight of the beans is not the same within a pod; the beans in the apical zone have significantly lighter beans than the beans in the other two zones (peduncular and median); it is the first time that this effect was studied and highlighted. As a result, resource partitioning is apparently not homogeneous within the pod, beans from areas further from the peduncle being smaller. The beans of clones with longer fruiting cycle durations are larger, i.e. the longer it takes for pods to mature, the larger their beans are. It was already observed for other fruits: the fruits with longer fruiting cycle durations are often larger^19,20^. In *T cacao*, although fruit development is dependent on climatic factors such as temperature^21^, an important genetic variation on the fruiting cycle duration was already found^22,23^. It was shown that the longer the fruiting cycle duration, the heavier the beans are. However, with a longer fruiting cycle, the greater the risk exposure of the pods to diseases such as black pod disease^24^ or frosty pod rot^25^. A trade-off has therefore to be found depending on the cultivation constraints of the areas.

The average weight of a bean is therefore a very heritable trait, but this trait is also dependent on several other characteristics: length of the fruiting cycle, number of beans per pod, location of the bean in the pods. The clones with Criollo ancestry had heavier beans (ICS 60, CCN 51). The length of the fruiting cycle of clones should be systematically characterized as it is a very heritable trait and explains several other traits of interest.
